# Collectanea

**Published:** 1830-05

**Authors:** 


### COLLECTANEA.

Floriferis ut apes in saltibus omnia libant,
Omnia nos, itidem, depascimur aurea dicta.

### PHYSIOLOGY.

*Communication between the Uterine and Placental Vessels.* Dr. BIANCINI published last year, in the Antologia Giorn. di So. &c., some experiments which he had made to prove that there exists a direct and immediate circulation between the mother and fœtus. He injected the vascular system of a woman who died in childbed, from inertia of the uterus, the placenta being still attached to the uterus: he found the injection in the vessels of the chorion and of the amnios, and, on examining the tortuous arteries of the uterus, he observed that they penetrated the tissue of the placenta, that they spread themselves over these vessels, and that they had deposited the injection in the cells described by HUNTER and MECKEL. In a young woman who died eight days after delivery, and in whom a small portion of the placenta still adhered to the uterus, the injection introduced into the uterine arteries not only passed from the uterus into the adherent portion of the placenta, but also spread itself into the cavities of the uterus and vagina, through the lacerated extremities of the vessels which Dr. B. names utero-placental arteries. On dissection, the vessels of the uterus and placenta were found filled with the injection. In a woman who died in childbed from metrorrhagia, the injection introduced by the aorta exhibited the tortuous arteries continuous with those of the uterus, and which, by their free and open extremities, had permitted the injection to transude on the internal surface of the uterus. It was, Dr. B. thinks, by these utero-placental vessels that the fluid in the first experiments passed from the uterus into the placenta. These vessels (according to Dr. B.) M. LAUTH, jun. has improperly named lymphatics.

These experiments have given rise to much controversy in Italy. Among others, Dr. RIGALLI has opposed to them some anatomico-physiological observations, founded upon a great number of experiments made by him in 1819

and subsequently upon a woman, and the females of different species of animals. In the greater number of cases, the injection of the vessels of the uterus did not pass into those of the placenta: if, in some cases, the injection did pass into the placental vessels, the experimenter is convinced that this happened either from the rupture of the vessels, or by absorption, which function it is known goes on some time after death. In fact, if the passage into the placenta of fluids injected by the uterus occurs when the experiment is made immediately, or very shortly, after death, it never takes place when the experiment is tried a sufficient time after death for all vitality to be extinct, and absorption to have ceased. Dr. Rigalli is, therefore, convinced that the apparent results obtained by Dr. Biancini were the effects either of absorption, a pathological condition, or rupture of the vessels; and denies that they at all prove the connexion between the uterine and placental vessels, or the direct communication between the mother and fœtus.

On the other side, M. Daliso Casabianca has sent to the Medico-Physical Society of Florence a memoir on this subject, in which he says that, assisted by M. Vinciguerra, and in presence of the most celebrated professors of anatomy and physiology of the university of Pisa, he has made eleven experiments upon animals, in which mercury injected by the arteries of the mother passed into the veins of the fœtus, and *vice versa*, without its being possible, in this double passage, to discover any extravasation of the injection in the intermediate tissue. He concludes, 1st, that the fluids injected by the arteries of the mother passed into the umbilical vein of the fœtus; 2d, that the same fluids introduced by the uterine veins flowed into the umbilical arteries; 3d, that these fluids have a free circulation from the mother to the fœtus, and from the fœtus to the mother, by two series of vessels, described by M. Biancini under the name of utero-placental arteries and placento-uterine veins.—*Journal des Progrès.*

---

*Case of Twins united by the Umbilicus.* By Joshua Martin, M.D. of Xenia, Ohio. (*West Med. and Phys Journal,* from *Far. Rec. and Xenia Gaz.*)

The wife of a gentleman of this vicinity was delivered on Saturday, the 29th of August, at the close of the eighth month of utero-gestation, of two living children, of ordinary size, who were attached together by a round substance of about three inches diameter, commencing at the ensiform cartilage, or lower end of the breast bone, and extending down the abdomen.

The superior part of the attachment was hard and cartilaginous, formed by the ensiform cartilage of the one extending across, and uniting with that of the other; below it was soft, and gave the sensation to the touch of a membranous sac, containing part of the abdominal viscera.

At the inferior part of the connecting medium, the skin was wanting; and at that point arose one umbilical cord, which served both children.

Anastomosis, or union of the superficial veins of the two children, could be distinctly perceived. They were both females, and in every respect natural, except that one had two thumbs on the left hand. I saw them about forty-eight hours after their birth; one of them had then been dead twelve hours, and the other died in a few hours afterwards.

The conformation of these children appears to have been precisely similar to that of the Siamese boys.

### PATHOLOGY.

*On various Appearances of the Tongue, considered in relation to Diagnosis.* By Dr. Piorry. (*Journ. Hebdom. de Med.* No. 60.)

When the pulse is strong, frequent, full, and firm, and the conjunctivæ, the cheeks, lips and pharynx, and gums, are red, the tongue presents the same colour in a less intense degree: the difference of tint being easily explained by its peculiar structure. After profuse evacuations of blood, and chronic diseases, all the tissues and the tongue become paler than natural. In many patients affected with clearly-marked acute gastritis, enteritis, or dysentery, with but little febrile reaction, the tongue is more or less pale. In traumatic fevers, acute pneumonia, without gastric symptoms, the tongue has sometimes a vermilion hue, at others it is of a deep red. After bleeding, it becomes pale, although the stomach and liver are consecutively affected. This colour of the tongue frequently exists only at its edges, the middle being covered with a mucous secretion of variable appearance; but, if this mucous covering is removed, the tongue will be found of a uniform colour. The point of the tongue frequently becomes red by the effort which the patient makes to thrust it from the mouth. When the muscles of the organ are relaxed, the redness disappears. Dryness of the lingual surface appears to arise from evaporation of the moisture which ought to lubricate it, and which is probably always secreted in sufficient quantity for that purpose. Every cause which obliges a patient to respire by the mouth tends therefore to render the tongue dry, and every thing which increases the current of air within the cavity of the cheeks produces the same effect. Accelerated respiration frequently gives rise to this phenomenon. The tongue is usually very dry in acute pneumonia, particularly when coryza accompanies the disease. Such is also the case in pleurisy. Fever, attended by rapid contractions of the heart, and consequently quickened breathing; affections of the liver, stomach, and peritoneum, obstructing the descent of the diaphragm, and accelerating respiration, must have the same effect.

Repeated experiments and observations of the saliva and mucus under the influence of heat, have convinced M. Piorry that the principal cause of the formation of the various secretions with which the tongue and teeth are covered, arises merely from the desiccation at different degrees of heat of the fluids which ought to moisten the tongue. To this cause, he adds, as contributing to the different colours of these secretions, the nature of the saliva and mucus of the mouth, which corresponds with the nature of the blood, and which contains some of the elements that are found in it. Thus, in diseases of the liver, all the solid tissues are tinged with yellow; some of the fluids, as the urine and perspiration, have the same appearance. It is probable, therefore, that the saliva and mucus contain a small proportion of the colouring matter, which gives to the tongue the tint it presents in such cases. Lastly, abstinence will produce, in a very short time, the formation of the different coverings of the tongue referred to; and, upon taking food, they will disappear still more rapidly.

---

*Local Effect of Poisons.* Of nervous impressions, without any visible organic change, few well-authenticated and unequivocal instances are known. Mr. Brodie mentions a good example in the effects of monkshood on the lips when chewed:* it causes a sense of numbness and tingling in the lips, lasting for some hours, and quite unconnected with any affection of the general nervous system. Another instance, which was mentioned to me by M. ROBIQUET, of Paris, occurs in the effects of the strong hydrocyanic acid: when its vapour was confined for some time in a glass tube, with a finger on each open end, M. Robiquet remarked that the point of each finger became benumbed, and remained so longer than a day. These are unequivocal instances of a purely nervous and local impression on the external surface of the body. The most unequivocal instance I know of a similar impression on internal parts, is a fact related by Dr. W. PHILIP with regard to opium.† When this poison was applied to the inner coat of the intestines of a rabbit during life, the muscular contractions of the gut were immediately paralysed, without the general system being for some time affected. The same effect has been observed by Messrs. MORGAN and ADDISON to follow the application of ticunas to the intestine:‡ an instant and complete suspension of the peristaltic movement took place whenever it touched the gut. A parallel fact has also been described by Dr. MONRO, secundus:§ when an infusion of opium was injected between the skin and muscles of the leg of a frog, that leg soon became palsied, while the animal was able to leap briskly on the other three. Analogous results have further been obtained with the prussic acid by M. COULLON.‖ He remarked, that when one hind leg of a frog was plunged in the acid, it became palsied in thirty-five minutes, while the other hind leg continued perfectly sensible and irritable. Sugar of lead probably possesses the same property.

These facts are important, because some physiologists have doubted whether there really exist any local impressions of a purely nervous nature, unconnected with organic change, and arising from the action of poisons. Yet the existence of impressions of the kind is necessary to the stability of the doctrine of the sympathetic operation of poisons, that is, of the transmission of their influence from organ to organ along the nerves. Nay, in the instance of very many poisons supposed to act in that manner, we must still further believe in the existence of primary nervous impressions, which are not distinguishable by any local sign whatsoever.¶—CHRISTISON *on Poisons.*

---

***Diseases whose Effects are apt to be mistaken for the Effects of Poison.***

1. *Distention of the Stomach.* Mere distention of the stomach from excessive gluttony may cause sudden death. Generally, indeed, the symptoms and appearances in the dead body show that death is the consequence of apoplexy;

* Philosophical Transactions, 1811, p. 186.

† Experiments on Opium, 1795, reprinted in his Treatise on Fevers, iv. 697.

‡ Essay on the Operation of Poisonous Agents on the Living Body, 1829, p. 63.

§ Edin. Phys. and Lit. Essays, iii. 311.

‖ Recherches sur l'Acide Hydrocyanique, 1819, p. 179.

¶ It is a singular fact, and well worthy of mention, that the poisons which unquestionably act on the sentient extremities of the nerves, and indirectly through the nerves on the brain and spine, do not act at all on the cut surface of the brain and nerves, or upon any part of the course of the latter. This has been proved with respect to hydrocyanic acid, opium, strychnia, and all active narcotics.—*Christison,* p. 22.

but sometimes not. In order not to break the continuity of my remarks on the diseases of the stomach which imitate poisoning, it may be useful to consider in the present place all the varieties of the effects of distention.

Excessive distention of the stomach, then, sometimes causes sudden death by inducing apoplexy, which is commonly of the congestive kind, that is, without rupture of vessels. MÉRAT has related an instructive case of this kind: A man in good health, while greedily devouring an excellent dinner, became suddenly blue and bloated in the face; a clammy sweat broke out over his body, and he died almost immediately. On dissection, the stomach was found enormously distended with food, and the vessels of the brain were so gorged, that the brain appeared too large to be contained within the skull.*

But there is reason to suppose that death from distention is the consequence not always of apoplexy, but sometimes also of an impression on the stomach itself. Sir EVERARD HOME relates the case of a child, who, being left by its nurse beside an apple-pye, was found dead a few minutes afterwards, and in whose body no appearance of note could be discovered, except enormous distention of the stomach with the pye. A still more distinct case in point forms the subject of a medico-legal report by WILDBERG: a corpulent gentleman died suddenly fifteen minutes after dinner, and, as he lived on bad terms with his wife, a suspicion arose that he had been poisoned. His wife said that he fell asleep immediately after dinner, but had not slept many seconds when he suddenly awoke in great anguish, called out for fresh air, exclaimed he was dying, and actually expired before his physician, who was instantly sent for, could arrive. Wildberg found the stomach so enormously distended with ham, pickles, and cabbage soup, that, when the belly was laid open, nothing could be seen at first but the stomach and colon. Some white powder was found on the villous coat of the stomach, and it was at first suspected to be arsenic; but it proved on analysis to be merely magnesia, which he was in the habit of taking frequently. The diaphragm was pushed high into the chest by the distended stomach. There was not any particular congestion in the brain. Wildberg very properly ascribed death to simple over-distention of the stomach.† In all such cases the symptoms may be suspicious; but when carefully considered, they can hardly be said to resemble closely the effects of irritant poisoning, and at all events the appearances in the dead body will at once distinguish them.

2. *Rupture of the Stomach* is not a common occurrence, but it sometimes imitates closely in its symptoms the effects of the irritant poisons.

It is generally the consequence of over-distention, combined with efforts to vomit. On account of the abrupt turn which the gullet makes in entering an excessively distended stomach, the cardia or opening of the gullet into the stomach becomes valved, and the contents cannot be discharged by vomiting. A striking case of this kind is related by Dr. LALLEMAND, in his Inaugural Dissertation at Paris in 1818: A woman, convalescent from a long attack of dyspepsia, being desirous to make amends for her long privations as to diet, ate one day to satiety. Ere long she was seized with a sense of weight in the stomach, nausea, and fruitless efforts to vomit. Then she all at once uttered a piercing shriek, and exclaimed that she felt her stomach tearing open:

* Dictionnaire des Sciences Médicales, art. Indigestion, p. 374.

† Praktisches Handbuch für Physiker, iii. 292.

afterwards she ceased to make efforts to vomit, soon became insensible, and in the course of the night she expired. In the fore part of the stomach there was a laceration five inches long, and a great deal of half-digested food had escaped into the cavity of the abdomen. The coats of the body of the stomach were healthy, but the pylorus, or opening into the intestines, was indurated, which had been the cause of her dyspepsia.

In other cases of death from rupture, the laceration is caused not by the accumulation of food, but by the accumulation of gases arising from depraved digestion, constituting a disease almost the same as that which so often attacks cattle that have fed on wet clover. A singular example of this rare affection, in which death was preceded by the symptoms of irritant poisoning, has been noticed by Professor BARZELOTTI.*

Another rare variety of rupture of the stomach must also be particularly noticed, because the course of the symptoms imitates very closely a case of poisoning with the irritants. It is *partial rupture*, or laceration of the inner coat only. A very interesting case of that description has been related by Mr. CHEVALIER: A youth of fourteen, on the evening after a Christmas feast, at which he ate and drank heartily, was attacked with violent and frequent vomiting. Next morning he said he felt as if the blood in his heart was boiling; he was unable to swallow, the pulse became irregular, and pressure on the heart or stomach caused excruciating agony. These symptoms continued till the following day, when he vomited two pounds of blood at successive intervals, and soon afterwards expired. The inner coat of the stomach was torn in many places, and that of the duodenum was lacerated almost completely round. No other disease existed either in the bowels or elsewhere.†

Some of the cases now mentioned could hardly, I apprehend, be distinguished from the effects of certain irritant poisons by the symptoms only; but the morbid appearances in the stomach will at once determine their real nature.

Rupture of the stomach, it should be observed, does not always occasion the symptoms hitherto related. Sometimes it causes instant death. Thus, a healthy coal-heaver in London, while attempting to raise a heavy weight, suddenly cried out, clapped his hand over his stomach, drew two deep sighs, and died on the spot. On dissection, a lacerated hole was found in the stomach, big enough to admit the thumb; and the stomach did not contain any food.‡ This case shows that rupture may take place without previous distention.

3. *Rupture of the Duodenum* is a very rare accident from internal causes. The following instance resembles considerably the symptoms of irritant poisoning: A gentleman, forty-eight years old, quarrelled violently with another while playing billiards immediately after dinner; soon afterwards he was seized suddenly with violent pain in the stomach, vomiting, cold extremities, failing pulse, and he died very soon. The mucous coat of the duodenum was found much inflamed, and four inches and a half from the pylorus there was a lacerated hole involving a third of the circumference of the gut.§—*Ibid.*

* Medicina Legale, ii. 22.
† London Medico-Chirurgical Transactions, v. 93.
‡ London Medical Repository, xvii. 108.
§ Bulletins des Sciences Medicales, x. 64.

*On certain Affections of the Bones of the Cranium.* A woman, aged forty-eight, about a year before her death, fell down a stair, and received various injuries, especially one on the head, which confined her to bed for some days. From this time her health was bad: she generally complained of fixed pain of the head, and had a very disordered state of the stomach and bowels. She was able, however, to attend to the ordinary duties of her family, till about three weeks before her death, when she was seized with fever and outrageous delirium. These symptoms subsided after a bleeding; and next day she had erysipelas of the face, which went off in a few days. She was then able to be out of bed, but complained of a fixed and deep-seated pain in the right side of the head, a little above the ear, and there was discharge of matter from the right ear. She continued in this state, sitting up part of every day, till three days before her death, when she became comatose, with partial paralysis of the left side, and frequent convulsive motions of the right arm. She died on the third day after the occurrence of these symptoms.

*Inspection.* The cranium was very easily opened, the bones being remarkably soft. On raising the skullcap, the inner surface of the whole upper part of the cranium exhibited a singular state of disease: The inner table seemed to be wanting through its whole extent, and there appeared the rough, irregular, and cancellated, structure of the central part of the bone. Betwixt this surface and the dura mater, there was a deposition of soft adventitious membrane of a yellowish colour, varying from one twelfth to one eighth of an inch in thickness. In raising the skullcap, this membrane in some places adhered to the dura mater, leaving exposed the irregular cancellated structure of the bone; and in other places it adhered to the bone, exposing the dura mater of its natural appearance. The parts affected by this singular state of disease were, the frontal bone above the orbitar plates, the whole of both parietal bones, the squamous portion of both temporal bones, and rather more than the upper half of the occipital bone. The greatest erosion was on the parietal bones, where several portions were very thin and transparent, and a few points were perforated. The external surface of the cranium was of a natural appearance, except at the few points where the erosion had perforated the bone by very small apertures. In the lower part of the right hemisphere of the brain, towards the posterior part, there was an extensive abscess. The brain in other respects was healthy. On the petrous portion of the right temporal bone, the dura mater was of a dark colour, and detached from the bone; but the bone was healthy.

I find no case described by any writer exactly resembling this remarkable affection of the bone. There was a complete destruction of nearly the whole inner table of the cranium; and in its place a deposition of a soft adventitious membrane, by which the dura mater was everywhere agglutinated to the diseased bony surface. This disease must have been going on for a considerable time; the abscess of the brain was probably recent, and the immediate cause of death. The patient was a respectable married woman, and there seemed no ground for suspecting a syphilitic taint: such a disease, therefore, is probably to be considered as the result of a slow inflammatory action affecting the bone, and gradually destroying it by caries. Such a disease may originate in an injury, or may commence without any obvious cause: it affects most commonly the external table of the skull, though it may likewise affect the internal; but I have seen no case described by any writer in which it was entirely confined to the internal table. A lady mentioned by Mr. HOWSHIP,* at the age of fifteen received a slight blow on the right side of the head, and for thirty years after was liable to severe headach, which was constantly referred to that spot. She then became drowsy, and her vision was impaired, and at the age of fifty she died comatose. At the seat of the original injury, the bone, to the extent of a crown piece, was so thin from absorption as to be almost transparent. The dura mater at this part was altogether removed, and the brain beneath was of a dark livid colour, and much indurated; and this disease extended through the whole middle lobe. In a case mentioned by DESAULT, death followed a blow on the head after a month; the bone was externally sound, and its coverings were healthy, but the internal table was blackened through the whole extent of one of the parietal bones; the dura mater adhered to the bone as firmly as to the other parts of the cranium, and there was suppuration on the surface of the brain.—ABERCROMBIE *on the Brain, &c. 2d edition.*

---

*De Hydrorrhea Uteri Gravidarum; Commentatio inauguralis.* Auctore JOANNES BAPTISTA GEIL, Heidelburg.

The affection treated of in this dissertation consists of a discharge of water from the gravid uterus, which is neither preceded nor followed by contractions, nor by dilatation of the orifice of the womb; disturbing utero-gestation very slightly or not at all, the woman going her full time, and always having the membranes distended in the usual manner during labour. Such discharges are rarer during the early stages of pregnancy, from the third even to the fifth or seventh month, and more common afterwards: the quantity of fluid is exceedingly variable, and sometimes an almost incredible discharge takes place. In some instances this occurs at a sudden gush: in others, the water dribbles away. The profluvium appears once or oftener, at different intervals, sometimes persisting for a long time, increased in quantity by violent excitement of body and mind, and the contrary; the quantity is sometimes greater at night than during the day. The cause of this condition is sometimes peculiar, and again is altogether undiscoverable. Some cases are preceded by no pain; others are preceded or followed by pains similar to those of labour. The fluid effused is generally yellowish, sometimes tinged with blood, and again perfectly limpid. In cases occurring near the full time, the fluid leaves rigid spots upon the clothes, and uniformly gives out that seminal odour peculiar to the proper fluids of the amnios.

Although this peculiar discharge has frequently been observed by very experienced men, it cannot be regarded as at all common; it might easily lead an inexperienced person to believe that abortion was about to occur, or that delivery must necessarily follow without delay. Yet it has happened that such discharges have occurred many days and weeks prior to delivery, and at the proper time the labour comes on, with properly distended membranes, as if no such effusion had taken place.

A great difference of opinion exists among accoucheurs concerning the source of these *false* or *spurious* waters, as they are called, to distinguish them from the waters within the fœtal membranes, whose displacement must necessarily be followed by parturition. Some think them to be contained

* Practical Observations in Surgery and Morbid Anatomy.

between the amnion and chorion, and to flow from a rupture of the latter; others, from a ruptured lymphatic, or a broken hydatid within the neck or body of the womb; others, from a transudation of fluid through the amnion, &c. The opinion first mentioned is the most generally received, because water is found between the chorion and amnion until the third month of pregnancy, and it is thought to be from its increase that such effusions arise. The writer objects to this mode of accounting for the fluid, that in various instances ova have been examined by himself and others, which had no intermembranous fluid. But we can scarcely attribute much importance to such facts, inasmuch as these ova were thrown off by abortion before examination, and might readily lose any such fluid, either by transuding from the chorion, or by endosmosis passing into the amniotic fluid.

From a view of all the facts, the author concludes that the discharge is from a fluid extravasated between the concave surface of the womb and the external or convex surface of the chorion. When this collection increases to a certain degree, it escapes from the orifice of the womb, in a mass and force proportioned to the operation of the excitement of body or mind which determines it. A clear distinction between discharge of these spurious waters and those from the amnion, is to be derived from the period of their occurrence, the absence of parturient efforts, and undilated state of the mouth of the womb, as well as the slight, if at all perceptible, change in the abdominal tumor of the patient. Such discharges should cause no alarm to the patient, nor provoke any interference from the midwife.

The causes of this singular flux of water, whatever may be the immediate point at which it is secreted, are to be sought for in the general condition of the patient, her mode of life, habit of body, or the state of the bowels, womb, and adjacent organs. Most commonly it is found to occur in females of rather robust and plethoric habit, in whom full diet and want of exercise determine the secretory irritation in an organ already under a powerful healthy excitement, and relief is readily obtained by reduction of diet and other antiphlogistic measures. The disease occurring in debilitated constitutions will, of course, require an opposite course of treatment.

Dr. Geil has appended to his dissertation several well-observed cases of this affection, occurring in the experience of Professor Naegele, who has also furnished him with a collection of references to high authorities who have witnessed and recorded such instances.

---

*Hydrophobia.* In Graefe and Walther's Journal, we find an account of some interesting experiments on rabid animals, by Dr. Hertwich. The following are the principal results:

1. Of fifty-nine dogs who were inoculated, fourteen became affected with real rabies.

2. In those cases where the inoculation failed, no assignable cause of the failure could be discovered. There exists, accordingly, a peculiar disposition for the virus of rabies, as for that of other contagious diseases. (A mastiff, four years old, went through regular series of experiments without any effect being produced; while seven other dogs, who were inoculated with him, and in the same manner, became rabid. Some dogs were several times inoculated before any contagion took place; in others, this effect was observed after the first experiment.)

3. It appears, accordingly, that in cases of doubtful rabies, one or two accidental or artificial inoculations are not sufficient to serve as negative proofs of the existence of rabies.

4. No communication of the disease ever took place by the perspiration; the contagious matter of rabies cannot, therefore, be of a volatile nature.

5. Its vehicle is not only saliva and the mucus of the mouth, but also the blood and the substance of the salivary glands. It does not appear to exist in the nervous pulp.

6. The power of infecting exists at every period of the confirmed disease, and even for twenty-four hours after the death of the animal.

7. The virus of rabies appears to be inactive if administered internally: of twenty-two dogs who were made to swallow it, none took the disease.

8. The application of saliva to fresh wounds appears to be as often followed by rabies as the bites of rabid animals.

9. It is, consequently, beyond all doubt that the disease is neither produced by the lesion, according to GIRARD's opinion, nor by the fear of the patient, as has been repeatedly asserted.

10. The opinion of BADER and CAPELLO, that in dogs who had become rabid from the bite of an animal primarily affected with the disease, the saliva did not contain the contagion; and that it existed only in primary rabies, has been proved, by several experiments, to be erroneous. (This particularly agrees with MAGENDIE's experiments, who, having inoculated a dog with saliva of a patient affected with hydrophobia, the animal became rabid after a month, and bit two others, who were also infected: from these last no further contagion was observed.)

11. During the period of the inactivity of the virus, there are no morbid alterations observable, either locally or in the general health of the dog thus infected, nor does the lower surface of the tongue ever exhibit vesicles. There exist, accordingly, no precursory symptoms, as in other contagious diseases.

12. The disease generally breaks out within fifty days after the inoculation, or the infliction of the wound: Dr. Hertwich never observed it occur at a later period.

13. Inoculation or infection from animals affected with fierce rabies very often produces the other modification of the disease, and vice versâ; they are, consequently, only different forms of one and the same disease.

14. It is an erroneous opinion that healthy dogs are able to distinguish those affected with rabies by the smell; this is not the case; nor do they abhor food mixed with the secreta or excreta of rabid dogs.

---

*Curious Case of Metastasis occurring in a Woman during Lactation.* By Dr. F. FRARCHINA. (*Giorn. Anal. di Med.* for December 1828, and February 1829.)

A nurse went to Milan for the purpose of getting a child to suckle; the following day, having obtained a male infant, she took it home with her. On her way, she nursed it several times, and also in the evening after her arrival at home; but, on the night following, she all at once felt as if a fluid descended from the breast to the abdomen, and shortly afterwards she voided by stools and urine a milky humor, which, according to the statement of the woman, presented all the characters of pure milk. This evacuation continued all night, to such an extent as to fill two urinals. In the mean time, and in one night, the milk disappeared from the breasts, so that it was no longer possible for the infant to get another drop. The following days these abnormal milky evacuations continued, but in less quantity. At length, after disappearing completely for twelve days, the milk returned to the breasts as regularly as before, and the woman resumed again the nursing of the child, which she had confided to the care of another nurse.

---

*Phlegmonous Abscess of the Prostate Gland.* By M. CHATEAUSUF. (*La Clinique.*)

A man, forty years old, complained for several days of pain in the perineal region, but principally near the anus. When he consulted M. C., he had almost an incessant desire to go to the water closet, and to make water, which he had great difficulty in passing. After some time it appeared that the stream of urine had overcome some obstacle, and the water passed freely, but caused in the passage a burning sensation in the urethra. M. C. introduced his finger into the rectum, and felt, through the anterior part of the intestine, a large hard tumor, which was painful to the touch. From the situation and feel of the tumor, it was evident that it was phlegmon of the prostate.

Leeches and emollient poultices to the part, the warm bath, and low diet, were ordered. In the course of a few days, pus was discharged by the anus and urethra, and the pain was subsequently much less. The urine was still voided with much difficulty. Intestinal gas and a worm were discharged from the urethra. The discharge of matter soon ceased. No bad symptoms afterwards appeared, and the patient was quickly capable of bearing laborious employment.

---

### PRACTICAL MEDICINE.

*Means of suspending the Secretion of Milk.* M. RANQUE, chief physician of the Hôtel Dieu of Orleans, employs with success, to diminish the sensibility of the mammary gland, upon which the secretion of milk depends, frictions morning and evening upon the breast with the following liniment: ℞. Laurel water ℥ij.; Sulphuric Ether ℥i.; extract of Belladonna ℈ij. He prescribes at the same time rigid diet and sudorific drinks.

M. R., it is said, employs this liniment with success in engorgements of the testicles, after using antiphlogistics.—*Journal des Progrès.*

---

*Treatment of Epilepsy.* By Dr. BORIE. (*Lancette Française*, Janv. 1830, No. 88.)

Considerable success appears to have been obtained from the following plan of treatment.

*Preparatory measures.* 1st. Two ounces of blood are to be taken from the feet. 2d. Four days after, a grain of Tartar emetic is to be given in any mild fluid. 3d. Four days after this, an ounce of castor oil in a vegetable broth. 4th. After the lapse of the same time, four grains of calomel are to be taken in a pill, with a cup of infusion of Polypodium filix mas.

*Treatment.* 1st. Twenty drops of distilled Lauro-cerasus are to be taken in sugar and water; the dose being increased one drop each day till it amounts to sixty, and then to be discontinued. 2d. At bedtime, two drachms of powder of Wormwood* in a glass of infusion of Linden wood. 3d. Every fifteen days, a moxa to be applied upon the spinal column, commencing on the cervical region. Six moxas will be sufficient in most cases. 4th. The patient constantly to wear a magnetic bracelet upon the left arm, which is to be firmly tightened upon the approach of a paroxysm. 5th. The lower extremities to be briskly rubbed with ether twice a day.

*Regimen.* 1st. To wear flannel habitually next the skin; to bathe in fresh or salt water, plunging in head foremost. 2d. Exercise in the open air, avoiding exposure of the head to the rays of the sun. 3d. To avoid all mental excitement and causes which are likely to debilitate the body, especially onanism or venereal indulgence. 4th. Diet, vegetables and water.

---

*Paralysis of the Face from an Affection of the Portio Dura, cured by Galvanism.* By M. H. Montault. *(Revue Med.)*

For some days the patient had experienced a lancinating and remittent pain behind the right ear, and during the last three days the tears were not absorbed by the lachrymal punctæ of the right eye, and flowed over the face. Shortly afterwards, paralysis of the right side of the face occurred. During the act of laughing, whistling, or masticating, the left commissure of the lips was always drawn upwards and outwards, whilst the right was distended and entirely passive. The right eye was constantly exposed, as the eyelids on that side could not be closed. The skin of the forehead and upper part of the nose, on the right side, was perfectly motionless, whatever grimaces the patient attempted to make. Neither feeling, sight, hearing, nor taste, were affected on either side. The cause of this attack could not be satisfactorily ascertained. From bleeding locally and generally, mustard sinapisms, stimulating enemas, fomentations, &c., and a strict attention to diet, no relief whatever was derived. In a week from the attack, the patient could neither articulate nor masticate.

MM. Salandière and Pichonnière were consulted, as they had paid much attention to the treatment of such diseases by means of electricity, galvanism, and acupuncture. They predicted a speedy cure. The part was electrified by sparks and shocks, and immediately afterwards four or five needles were introduced; one at the exit of the facial nerve, and others upon its branches. Two of the needles were connected, by means of a metallic conductor, with one of the poles of a galvanic pile of thirty plates. The three other needles were made to communicate, by the same means, with the opposite pole. When the pile was charged with nitric acid, severe pains and slight motion were produced; bnt, when charged with hydrochloric acid, the sensation caused was much less acute, and the muscular contraction was the principal phenomenon which followed. The patient submitted to this discipline for seven days; from twenty minutes to half an hour each time. In a few days he was entirely cured: the natural appearance and action of the parts were perfectly restored.

* It is upon this part of the treatment the author chiefly relies. In Sweden and other parts of the continent, wormwood is considered a specific against epilepsy. We have tried it in two cases, which appeared favorable for such a remedy, without any effect.—Editor.

*On the Use of Acetate of Lead in Ulcerated Phthisis.* Dr. LENZ considers the acetate of lead as a true panacea in chronic pneumonia which was gone on to ulceration. Dr. Schneider, of Ettenheim, is also said to have employed the remedy in this disease, with success. The medicine is given in powder, combined with opium; the dose gradually increased. A patient who was cured by Dr. Lenz, took two drachms in thirty-two days; and Dr. Schneider has often given fourteen grains in one day, without producing the least ill effects.—*Heidelb. Klinische Annalen.*

---

*Warts cured by the Decoction of Tormentilla.* Mr. TYRRELL has for two or three years been employing a decoction of tormentilla root, as an application to the warts so common and usually so troublesome about the glans penis and prepuce. The form is as follows: R. Pulv. Radicis Tormentillæ ℥j.; Aq. ferventis ℔ss. dein cola. The surface of the affected part should be well cleansed, three or four times a day, with tepid water, and otherwise kept constantly covered with a piece of lint saturated with the decoction.—*Med. Gazette.*

---

*Case of Bronchocele relieved by Iodine. Effects of this Medicine on the Genitals.* A young man, æt. eighteen, of a lymphatic constitution, had, from his fifteenth year, when he attained to puberty, been affected with bronchocele, which soon reached such a size as to produce considerable dyspnœa, and frequent attacks of suffocation and hoarseness. Being admitted into the Hôtel Dieu, the tumor was found so large as to occupy the whole space between the middle of the neck and the clavicles; it was formed of two lobes, and was lifted up by the pulsation of the carotids; in its substance, also, an alternating enlargement was visible during the arterial expansion. The general health of the patient not being affected, he was put under a course of iodine, of the tincture of which he took from six to ten drops daily. The tumor gradually subsided, its lobes became more distinct, the voice more natural, and the difficulty of respiration ceased altogether. It was worthy of remark, that, under the use of iodine, the genitals became, as it were, atrophic; and that erections and pollutions, to which the patient had formerly been very subject, were never observed during this time.—*La Clinique.*

---

### SURGERY.

*Complete Amaurosis cured by the Extraction of a diseased Tooth.* (GRAEFE, *Journ. der Chirurgie, &c.* vol. xii. cah. iv.)

F. Przesmycki had always enjoyed good health, with the exception of occasional attacks of rheumatic headach and pains in the joints. In the autumn of 1825, he was suddenly attacked with shooting pains in the upper jaw of the left side, which extended to the eye. These violent pains, which were supposed to arise from cold, lasted several days, and then subsided; but returned periodically. A short time afterwards the pain returned with more violence, and again were incessant. The sight of the left eye was completely destroyed. Various modes of treatment were applied without benefit, and after eight months the left cheek had swollen; and, one night, several spoonsful of bloody pus was discharged from between the conjunctiva and the eyelids of the left eye. The pain now diminished, but extended to the temple; blindness continued. At the expiration of three weeks there was another discharge of pus, and it was occasionally repeated during the next six months.

Professor Galenzowki was now consulted. He conceived that the pus had formed in the maxillary sinus, and made its way along the orbitar part of the superior maxillary bone; but knowing also that suppurations of the upper jaw frequently depend upon carious teeth, the mouth of the patient was carefully examined, and a rotten tooth was found corresponding to the antrum of Highmore. This tooth was extracted to give exit to the matter; and, much to the astonishment of the surgeon, there was found fixed to its root a splinter of wood, about three lines long, and as thick as the head of a pin. A probe introduced through the alveolar process penetrated into the sinus, and gave issue to a few drops of pus. In nine days the patient entirely regained his sight, after having been deprived of it for more than eighteen months.

---

*Excision of the Tonsils.* Catherine Cunningham, æt. twenty-four, a servant in town, was admitted on 26th August on account of sore throat and deafness. On examination I discovered a very large swelling of the tonsils, which sufficiently accounted for both her complaints. I cut away the projecting portion on both sides by means of a hook and scissors, immediately after which she recovered her hearing, and ceased to be troubled with the uneasy symptoms in her throat.

Chronic enlargement of the tonsil is a very common disease in cold moist countries such as this, and the inconvenience which proceeds from it is no less complicated than distressing. The faculties of speech, smelling, tasting, and hearing, are all more or less affected, besides the constant uneasiness which is caused by the presence of a hard swelling in the throat, and the frequently recurring annoyance of inflammation in the morbid growth. It is therefore a fortunate circumstance that surgery affords a ready and certain mode of relief, viz. excision of the tumour. In doing this it is not necessary to remove the whole of the swelling, since the new actions, which are induced by the abstraction of a part of it, generally suffice for curing the remainder. The operation, therefore, is perfectly safe and easy; indeed, even granting that it were necessary to remove the whole of the tumor, the operation would still be attended with hardly any danger, since the carotid artery, which is the grand source of apprehension, runs parallel with the direction in which the incision ought to be made.—*From* Mr. Syme's *Quarterly Report of the Edinburgh Surgical Hospital.*

---

*Fracture of the Leg, treated by* Baron Larrey's *Plan.* M. Larrey lately presented to the Royal Academy of Medicine a hussar who had fallen from his horse, and broken both bones of his leg, and luxated the first metatarsal bone. The skin of the sole of the foot was lacerated, and the metatarsal bone projected through the wound. Amputation appeared almost inevitable; but M. Larrey determined to avoid, if possible, so serious an operation. He extended the wound, removed the metatarsal bone, and then brought the edges of the wound together, and covered it with a compress and pledgets dipped in a balsamic ointment; after which he reduced the fracture of the leg, and placed it in his apparatus without splints. The wound of the foot was not dressed for twenty-eight days, and the leg was not touched for fifty-one days. At the end of this time the parts were perfectly united; the foot had recovered its natural shape, with the exception of the depression which was the necessary consequence of the removal of the metatarsal bone. The motions of the foot were not perfect, but the man was daily recovering the natural use of the limb.

### MATERIA MEDICA.

*On the Effects of Camphor in a Healthy Individual.* By Dr. LUCAS SCUDERY, of Nessina. (*Annali Universali di Med.*)

It is extremely difficult, from the difference of action of the same medicine upon different individuals, the age, sex, constitution, &c. of the individual influencing the effects, to arrive at any general results. The following experiments on camphor appear, however, to have been made with great care; they were performed in the presence of several persons, who themselves submitted afterwards to experiment; so that confidence may be reposed in the results.

From ten to fifteen grains of camphor taken in the stomach, produces in from fifteen to twenty minutes a decided acceleration with increased force of the pulse, which continues permanent for one or two hours. There are produced, at the same time, redness of the face, dryness of the skin, headach with vertigo, increased sensibility to light, and brightness of the eyes; injection of the conjunctiva; stricture of the chest; odour of camphor in the breath; desire to urinate; urine smelling of camphor, passed in small quantities, and producing a burning in the urethra; constipation. These symptoms generally disappear at the end of about four hours; during the night the sleep is disturbed by voluptuous reveries, with erection of the penis, and pollutions. These effects, which were produced in five successive experiments, were more decided when the camphor was taken dissolved in alcohol; their intensity was also in proportion to the increase of the quantity taken; its effects were also then more prolonged, and accompanied with feverishness. M. S. has swallowed progressively as much as two scruples of camphor at a time, in many experiments. Drs. Pasquali, Mazzetti, and Gassoni, repeated these experiments, and with the same results.

A month after these experiments, Dr. Scudery wishing to ascertain what modification nitre would produce in the action of camphor, swallowed two scruples of the latter article, and five minutes afterwards he took two drachms of nitre dissolved in water. He experienced almost immediately afterwards nausea, with chilliness, and an abundant salivation. In about ten minutes the pulse lessened, but before twenty-five minutes, it had acquired new force; there supervened slight headach, confusion of ideas, the pain in the head gradually augmented, the light appeared stronger than natural, and objects clearer; the conjunctiva was injected; face warm; desire to urinate; pulse vibratory and frequent. In this state, Dr. S. took another solution of two drachms of nitre: immediately afterwards the nausea reappeared, but the headach and other phenomena disppeared: after some minutes it reappeared, but slightly. Half an hour afterwards, he passed a small quantity of urine with pain; pulse natural; during the remainder of the day no phenomena appeared, he passed a calm night; urine abundant, and depositing a sediment; he had two alvine evacuations. These experiments like the preceding, were frequently repeated with different doses of nitre and camphor; and Mr. Mazzetti also made some trials with them.

From all these experiments, Mr. Scudery concludes, 1st. That, in the dose of from eight to ten grains, camphor produces in a healthy man scarcely any appreciable effect; and that in diseases it should be given to the extent of two scruples, but in divided doses. 2d. That one of the effects of camphor upon the system, is to produce an excitation, characterised by an acceleration of the circulation, and an elevation of animal heat. 3d. That it produces no irritation of the gastro-intestinal mucous membrane; that it excites neither pain nor porporygmus, but that it constipates. 4th. That it acts specially upon the genito-urinary organs, augmenting the energy of their functions; voluptuous reveries, erection of the penis, sensation of heat in the urethra when urine is voided, are proofs of its stimulating action. 5th. That vertigo, the vivid impression of light on the eye, headach, acceleration of the circulation, and excitation of the genito-urinary organs, &c. announces that camphor acts directly upon the brain and the great sympathetic. 6th. That the stimulating effect of camphor is augmented by uniting it with another stimulant, as alcohol; whilst nitre, on the contrary, diminishes its stimulating properties.

---

### MIDWIFERY.

*Gestation prolonged beyond the ninth Month.* Dr. ALBERT, of Wiesentheid, has published in the *Zeitschrift für die Staatsarzheykunde*, iii. tes 1828, an account of two cases of this kind. In one, gestation was prolonged forty-three, and in the other thirty-three, days beyond the ordinary period.—*Arch. Gen.*

---

### MISCELLANEOUS.

*Prize Question.* The Medical Society of the department of the Seine propose the following question. Memoirs to be addressed "dans les formes académiques," to M. Nacquart, secretary to the Society, rue Saint-Avoye, No. 39.

To determine, by clinical observation, by experience from morbid anatomy, or by a series of experiments, the condition of the blood in diseases: to indicate the primary and secondary alterations of which the blood is susceptible, and the influence which they respectively exert in diseases.

As the Society are perfectly aware of the difficulties of the subject, it is determined to admit papers which treat only of one part of it; e. g. a description of any one kind of alteration of the blood in any one disease. Such communications will either be allowed to share the prize, or be recompensed with medals, according to their merits.

---

*Buccina; new Principle in Box-wood.* An apothecary of Bordeaux announced to the Pharmaceutical Society of Paris, at its last sitting, that he had discovered in the wood, and particularly in the bark of the box tree, an alkaline principle, to which he gives the name of Buccina. It is in the form of powder, and neutralizes acids, forming uncrystallizable salts: it has a very strong sudorific action and bitter taste. M. DUPETIT THOUARS, in making this statement at the Philomathic Society, remarked that buccina might perhaps be advantageously used in the manufacture of beer, "for there is more box-wood than hops employed in making almost all the beer brewed in Paris."—*New Mon. Mag.*

*Another Duplex Child.* Mr. Wm. Morgan, m.r.c.s.l., of Carnarvon, has published, in the last Number of the Medical Gazette, an account of a poor woman, the wife of W. Thomas, a quarryman, residing near Llanberis, in his neighbourhood, who was, on the 28th of March last, delivered of a duplex female child, still-born, which had two heads, two necks, four arms, two pelves, four thighs and legs, all perfectly and naturally formed. The two trunks were united at the breast-bones, and the union extended as low as the umbilicus, where it terminated in one umbilicus common to both. Their union was so remarkably firm that, from the examination, it appeared to be of bone: suffice it to say that, at their union, Mr. Morgan could not separate them the space of a line. It appears evident that their faces, and the fore parts of their bodies, were fronting each other. The midwife in attendance reports that they were born with their arms around each other's necks. From a careful external examination, (for Mr. Morgan was not permitted to make any other,) there appeared no reason why they should not have lived. It was evident they were of the full growth of the usual term of utero-gestation. The mother fancies they perished about a week before her accouchement. They measured in length seventeen inches and a half, and were supposed to weigh about nine pounds. Their features much resembled each other, but the hair of the head of one was somewhat darker than that of the other.

---

*On the Presence of Hydrocyanic Acid in the Human Body.* It is well known that hydrocyanic acid exists in many vegetables; that it is contained in the essential oil of the laurel, bitter almond, &c. It yet remains to be positively determined whether the animal body also contains this principle, either in a state of health or disease. From some experiments, which are still imperfect, it would appear that this fact is not improbable. Many observers have declared that they have detected this acid in the organic structure of some of the inferior animals; and Tiedemann and Gmelin have discovered it in the saliva of a man, and in that of a sheep. Woehler also concludes from his experiments, that urea is composed of a cyanate of ammonia.

---

